# Selection and Outcome of Portal Vein Resection in Pancreatic Cancer

**DOI:** 10.3390/cancers2041990

**Published:** 2010-11-24

**Authors:** Akimasa Nakao

**Affiliations:** Department of Surgery II, Nagoya University Graduate School of Medicine, 65 Tsurumai-cho, Showa-ku, Nagoya 466-8550, Japan; E-Mail: nakaoaki@med.nagoya-u.ac.jp; Tel.: +81-52-744-2233; Fax: +81-52-744-2255

**Keywords:** pancreatic cancer, portal vein resection, isolated pancreatectomy, catheter-bypass of the portal vein

## Abstract

Pancreatic cancer has the worst prognosis of all gastrointestinal neoplasms. Five-year survival of pancreatic cancer after pancreatectomy is very low, and surgical resection is the only option to cure this dismal disease. The standard surgical procedure is pancreatoduodenectomy (PD) for pancreatic head cancer. The morbidity and especially the mortality of PD have been greatly reduced. Portal vein resection in pancreatic cancer surgery is one attempt to increase resectability and radicality, and the procedure has become safe to perform. Clinicohistopathological studies have shown that the most important indication for portal vein resection in patients with pancreatic cancer is the ability to obtain cancer-free surgical margins. Otherwise, portal vein resection is contraindicated.

## 1. History of Portal Vein Resection

The procedures for pancreatoduodenectomy (PD) and alimentary tract reconstruction after PD were established during the 1940s [1,2,3]. PD became the treatment of choice for cancer of the pancreatic head region. The importance of combined resection of the portal vein for pancreatic head cancer to increase resectability and radicality was emphasized by Child [4]. He performed a two-stage operation. The first stage involved ligation of the portal vein; then, after development of collateral circulation, PD combined with portal vein resection was completed as the second stage, without reconstruction of the portal vein. However, this two-stage operation had a definite disadvantage; therefore, it was never further developed [4]. One-stage PD combined with portal vein resection using portocaval anastomosis was performed by McDermotte [5], but this procedure was not pursued either because of the possibility of Eck syndrome. Therefore, reconstruction of the portal vein is necessary. To reconstruct the portal vein after resection, homo- or autograft vessel transplantation [6,7,8] and the use of an artificial vessel [9,10] have been reported. The ideal reconstruction of the portal vein is end-to-end anastomosis of the portal vein [11,12,13]. This procedure has become quite common. The catheter-bypass procedure of the portal vein has since been developed and has contributed to portal vein resection and reconstruction with safety and ease [14]. Using this catheter bypass procedure of the portal vein, isolated PD combined with portal vein resection has been performed, which involves a non-touch isolation technique [15].

## 2. Catheter-Bypass Procedure and Isolated Pancreatectomy

**Figure 1:**
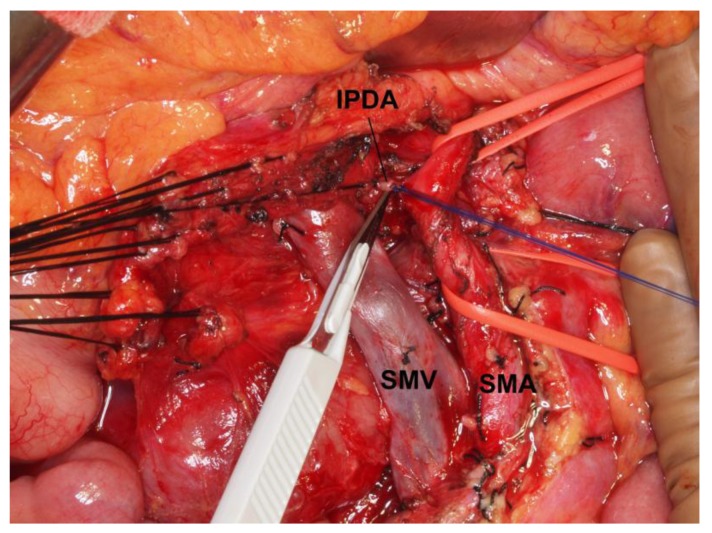
Photograph of lymph node dissection around the superior mesenteric vein and artery, by the mesenteric approach. The inferior pancreatoduodenal artery is exposed, ligated and divided. SMA, superior mesenteric artery; SMV, superior mesenteric vein; IPDA, inferior pancreatoduodenal artery.

**Figure 2:**
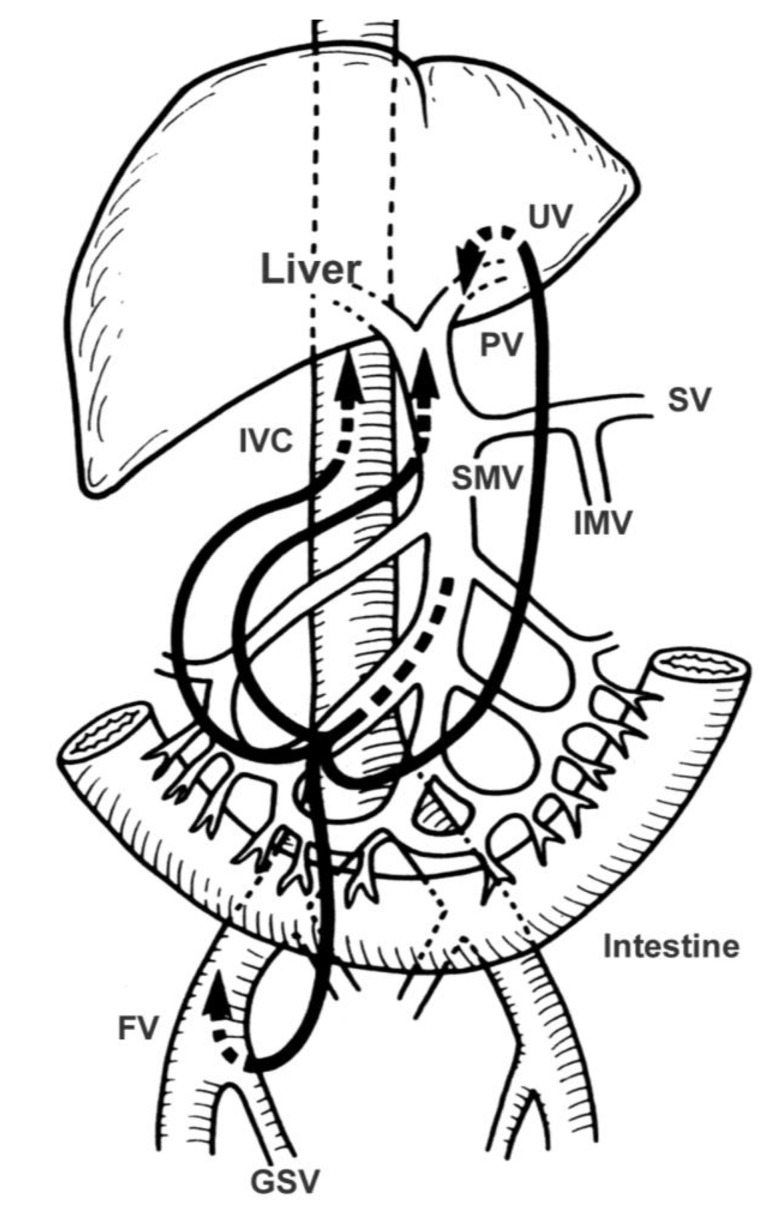
Procedures for bypassing the portal vein. UV, umbilical vein; PV, portal vein; SV, splenic vein; SMV, superior mesenteric vein; IVC, inferior vena cava, IMV, inferior mesenteric vein; FV, femoral vein; GSV, greater saphenous vein.

**Figure 3:**
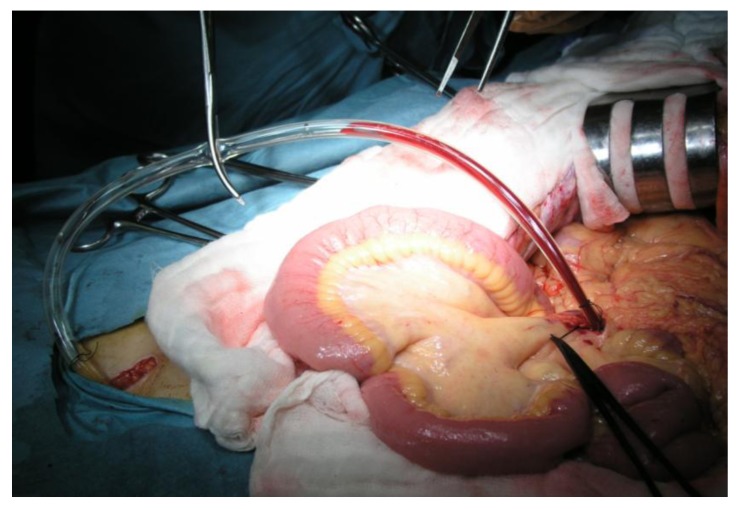
Photograph of catheter-bypass between the mesenteric and femoral veins. One end of the catheter is inserted in one of the branches of the superior mesenteric vein, and the other end in the femoral vein via the right greater saphenous vein. Portal venous blood flows into the femoral vein owing to the pressure differences between the portal and femoral veins.

**Figure 4:**
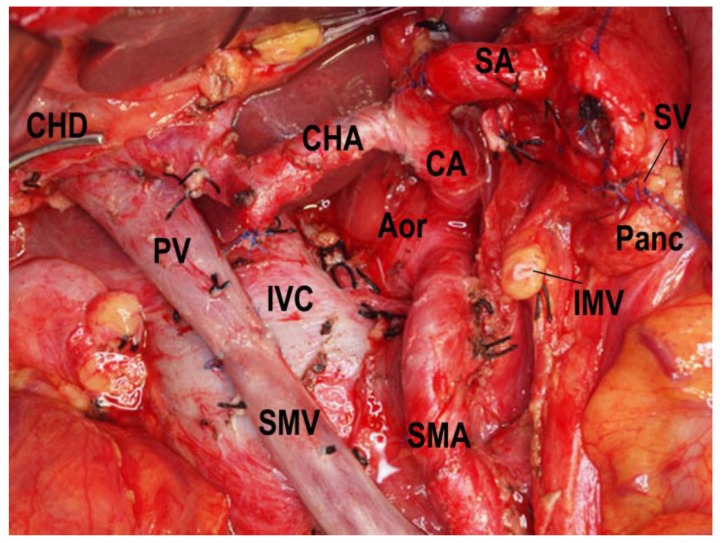
Isolated PD combined with portal and superior mesenteric veins resection, para-aortic lymph node dissection, and reconstruction of the portal vein by end-to-end anastomosis is done under catheter-bypass of the portal vein. PV, portal vein; SMV, superior mesenteric vein; CHA, common hepatic artery; SA, splenic artery; SV, splenic vein; CA, celiac artery; Aor, aorta; IMV, inferior mesenteric vein; Panc, pancreas; CHD, common hepatic duct; IVC, inferior vena cava; SMA, superior mesenteric artery.

In PD, the arteries that flow into the pancreatic head region are ligated and divided, and the drainage veins from the pancreatic head are ligated and divided before manipulation of the pancreatic head. Kocher’s maneuver is not performed in isolated PD. The first step of this operation uses a mesenteric approach to dissect lymph nodes and nerve plexuses around the superior mesenteric artery, and the inferior pancreatoduodenal artery is ligated at the root (Figure 1). Catheter-bypass of the portal vein using an antithrombogenic catheter was used to prevent portal congestion or hepatic ischemia during resection and reconstruction of the portal vein or simultaneous resection of the portal vein and hepatic artery (Figure 2, Figure 3) [14,15]. Para-aortic lymph node dissection is performed after isolated pancreatectomy and before reconstruction of the portal vein. Portal vein reconstruction is done by end-to-end anastomosis between the portal and superior mesenteric veins. No reconstruction of the splenic vein is necessary by distal gastrectomy (Figure 4).

## 3. Morbidity and Mortality

The morbidity rate of PD with portal vein resection has remained relatively high, whereas mortality rates of PD with portal vein resection have decreased. Siriwardana *et al*. have reviewed the outcome of portal vein resection during pancreatectomy for cancer [16]. They studied 52 non-duplicated papers that have provided relevant data from 1646 patients [16]. Data were available on operating time in 20 studies with a total of 616 patients. Histological evidence of portal vein invasion was detected in 668 (63.4%) of 1054 portal vein resection specimens. The rates of invasion ranged from 3% to 86% in 30 studies. Resection margins were positive in 346 (39.8%) of 870 patients with portal vein resection in 23 studies, with a range of 0–85%. Postoperative morbidity ranged from 9% to 78%, with a median per cohort of 42%. There were 73 (5.9%) reported deaths among 1235 patients in 39 studies that reported mortality after portal vein resection. The reported mortality rates in these studies ranged from 0 to 26%. The mortality rate of portal vein resection was >20% at the beginning of the era of portal vein resection 30 years ago; however, the rate has decreased to <5% in recent years.

## 4. Survival

**Figure 5:**
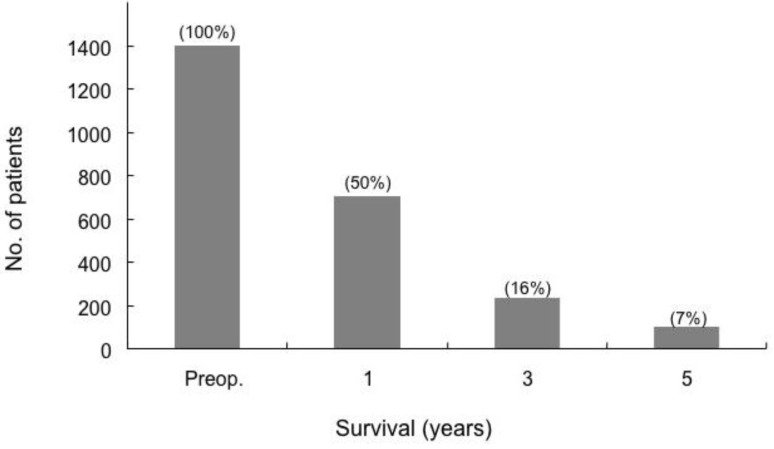
Survival after pancreatic cancer with portal vein resection. The blocks represent the total numbers of known survivors at each time interval (from [16]).

**Figure 6:**
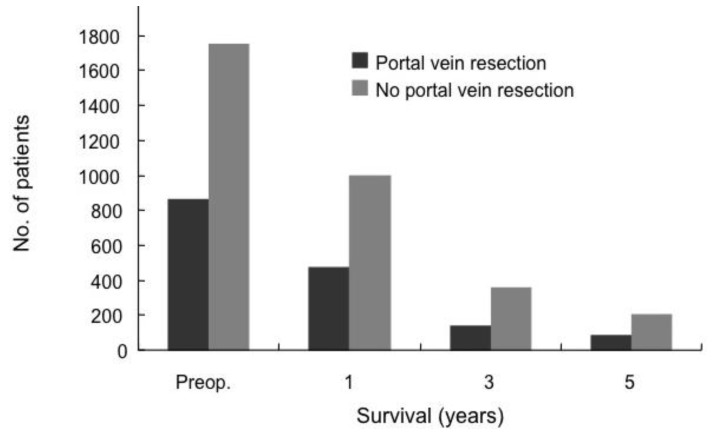
Comparison of survival in patients with or without portal vein resection (PVR). The blocks represent the total numbers of known survivors at each time interval. Comparisons were drawn by pooling data from 23 studies that had outcome data for pancreatectomy with portal vein resection. Note that this is not a parallel comparison of pancreatectomy with PVR in patients with tumor involvement *versus* pancreatectomy without PVR with tumor involvement, and patients without PVR are likely to have had earlier stage disease (from [16]).

**Figure 7:**
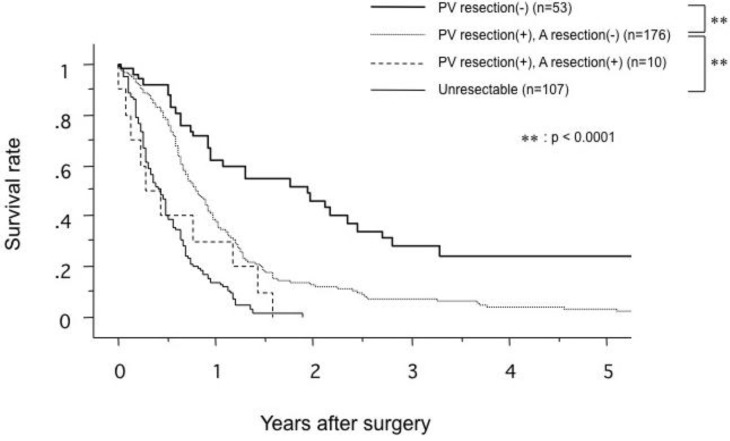
Comparison of cumulative survival rates in patients with no portal vein resection (PV resection(-)), portal vein resection (PV resection(+)), combined resection of portal vein and artery (PVAR; PV resection(+), A resection(+)), and unresectable pancreatic head carcinoma (from [19]).

**Figure 8:**
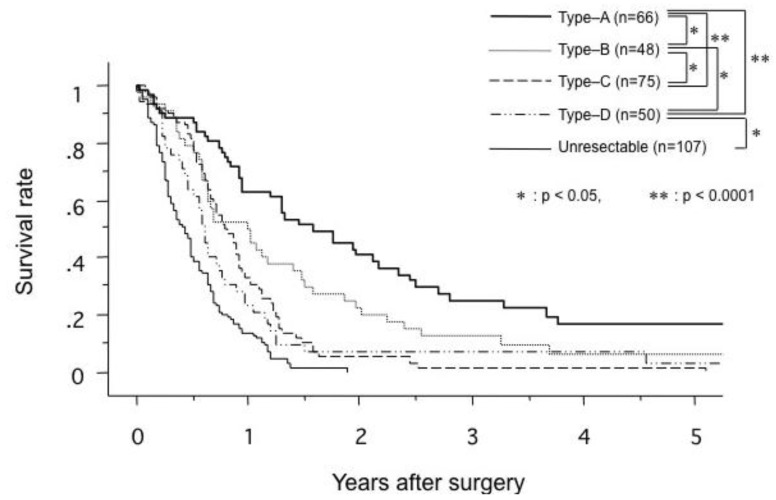
Comparison of cumulative survival rates according to the angiographic type of portography in patients with carcinoma of the pancreatic head. Type A, normal; type B, unilateral narrowing; type C, bilateral narrowing; type D, marked stenosis or obstruction with collateral veins (from [19]).

**Figure 9:**
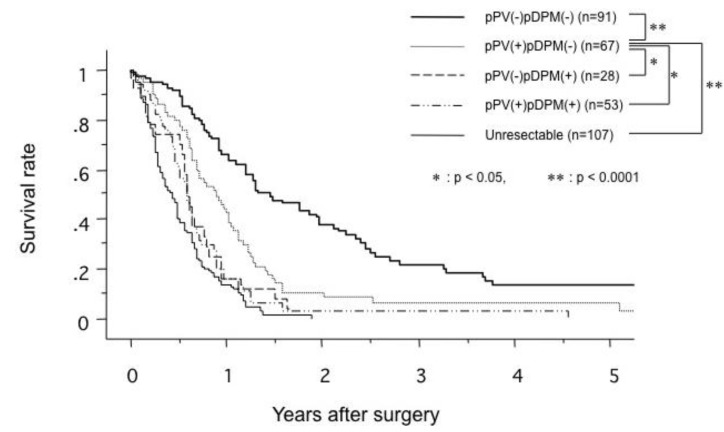
Comparison of cumulative survival rates in patients with and without histological invasion of a venous wall in the portal system (pPV) and invasion of the dissected peripancreatic tissue margin (pDPM) in patients with carcinoma of the pancreatic head (from [19]).

Siriwardana *et al*. have studied survival after portal vein resection during pancreatectomy for pancreatic cancer [16]. The median survival was 13 months for 917 patients who underwent portal vein resection in 31 studies. The reported median survival ranged from one to 109 months [16]. The one-, three- and five-year survival rate for 1,351 patients who underwent portal vein resection in 40 studies was 50%, 16% and 7%, respectively, as shown in Figure 5 [16]. Comparative survival curves from 23 studies of pancreatic resection with and without portal vein resection are shown in Figure 6 [16]. From 1981 to 2005, of 464 patients with pancreatic carcinoma, 305 (65.7%) underwent tumor resection in our department and vascular resection was performed in 212 (69.5%) of these. Operative mortality was 3.6% (11/305) in resected patients, 1.1% (1/93) in patients without vascular resection, 2.5% (5/197) in patients with portal vein resection without arterial resection, and 35.7% (5/14) in patients with portal plus arterial resection [17,18]. Figure 7 shows the cumulative survival rates, including operative and hospital deaths among patients with and without portal vein preservation, those with combined portal and arterial resection, and those with unresectable carcinoma of the pancreatic head. There was no significant difference in survival between unresectable patients and those who underwent combined portal and arterial resection. These data mean that carcinoma invasion to the superior mesenteric, celiac and common hepatic arteries is a contraindication for resection. Angiographic findings on portography were classified into four types: A, normal; B, unilateral narrowing; C, bilateral narrowing; and D, marked stenosis or obstruction with collateral veins [17]. Figure 8 shows that the prognosis after resection correlates with the angiographic findings in patients with pancreatic head carcinoma [17,18,19]. Cumulative survival rates based on histopathological portal invasion or invasion of the dissected peripancreatic tissue margin in resected pancreatic head cancer are shown in Figure 9. Histopathological carcinoma invasion of the portal vein wall was detected in 64.5% (12/186) in patients with portal vein resection for pancreatic head cancer. Survival for more than one year after resection was observed in the group with tumor-free margins, even when the portal vein wall had been invaded. In contrast, cumulative survival rates in patients with cancer-positive margins were quite low, and showed no statistically significant difference from the rate in patients with unresectable tumors.

## 5. Indications for Portal Vein Resection

**Figure 10:**
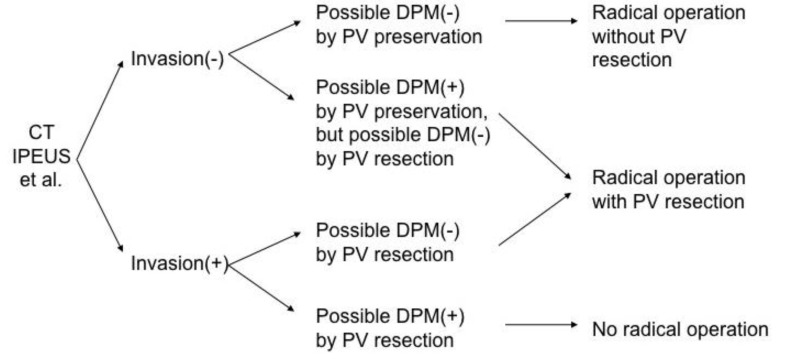
Indications for portal vein resection for pancreatic carcinoma.

Indications for portal vein resection in pancreatic cancer and criteria for resectability of pancreatic cancer are shown in Table 1. Preoperative staging, including portal vein invasion, for pancreatic cancer is usually performed with dynamic-phase spiral computed tomography, and intraportal endovascular ultrasonography also provides important information during surgery [20,21]. The algorithm for the indications for portal vein resection for pancreatic cancer is shown in Figure 10. Portal vein resection is indicated when carcinoma-free surgical margins are obtained by portal vein resection. There is no indication for portal vein resection in patients in whom surgical margins would become cancer-positive if such an operation were done. The safe operative procedure without intraoperative or postoperative complications is essential, and postoperative quality of life and social activity must be guaranteed.

**Table 1:** Criteria for resectability (from [19]).

**Resectable**
No distant metastases (liver, peritoneal, *etc*.)
No superior mesenteric, celiac or hepatic artery encasement
Normal portography
**Locally advanced resectable (Borderline resectable)**
Abnormal portography, but possibility of reconstruction
Tumor abutment on celiac or superior mesenteric artery
Invasion of stomach, colon or mesocolon
**Unresectable**
Distant metastases (liver, peritoneal, *etc*.)
Superior mesenteric, celiac, or hepatic artery encasement
Lymph–node metastases outside the dissection field
Portal or superior mesenteric venous invasion with obstruction indicating impossibility of reconstruction
Severe concomitant disease

## 6. Effect of Clinical Volume

For PD, several studies have reported the effect of institutional volume on patient outcomes. In 1993, Edge *et al*. [22] assessed 223 PD procedures from 26 university hospitals in the United States. The operative mortality was 6% (13/223) and the rate of severe complications was 21%, but they found that the caseload did not correlate with mortality. However, surgeons who performed fewer than four resections per year had more complications than those who performed more than four. In 1995, Lieberman *et al*. [23] assessed 1972 pancreatectomies including total pancreatectomies in 184 institutions in New York State. High-volume centers with more than 40 cases per year had significantly less mortality than low-volume centers (4% *vs*. 12.3%). Several other studies have also reported decreased mortality, length of hospital stay, and overall cost at high-volume centers compared with low-volume centers [24,25,26]. The definition of high and low volume varied among all these studies. Birkmeyer *et al*. [27] have reported a marked difference in mortality rates of PD in very low-volume (0 or 1 per year) and low-volume (1 or 2 per year) hospitals compared with higher-volume hospitals (>5 per year). In-hospital mortality rates at very low- and low-volume hospitals were significantly higher than those at high-volume hospitals (16% and 12%, respectively, *vs*. 4%; p < 0.001). These data strongly suggest that pancreatic resections should be done at institutions that perform a large number of them annually. In pancreatectomy combined with portal vein resection, more skillful technique, abundant experience and special postoperative care are necessary compared with PD without portal vein resection. Therefore, these types of operations should be done at large-volume centers.

Over the past 30 years, the operative mortality rate of pancreatectomy combined with portal vein resection has greatly decreased, and portal vein resection in pancreatic surgery has become a well-tolerated operative procedure in large-volume centers. The resectability rate of pancreatic cancer has increased by aggressive surgery combined with portal vein resection; however, the five-year survival rate is still low. Portal vein resection has been done in locally advanced cases of pancreatic cancer; therefore, a high incidence of cancer-positive surgical margins has been observed. Some patients with portal invasion who survive for more than years after surgery have been observed, and they are restricted within the state of cancer-free surgical margins. These findings show that portal vein resection is indicated when carcinoma-free surgical margins are possible. Therefore, preoperative and intraoperative diagnosis of cancer development is very important to decide the indications for resection of pancreatic cancer. These types of operation must be performed at large-volume centers.

## 7. Conclusions

Portal vein resection will be performed more often, safely and aggressively over the next five years if a cancer-free margin is obtained by resection. In addition to radical surgery, adjuvant therapy combined with chemotherapy, chemoradiotherapy and molecular targeting therapy might serve to improve the prognosis of pancreatic cancer.
